# Editorial: Hot topics in cellular neuropathology, volume II: promoting neuronal plasticity in the injured central nervous system

**DOI:** 10.3389/fncel.2023.1269763

**Published:** 2023-09-04

**Authors:** Dirk M. Hermann, Marco Bacigaluppi, Luca Peruzzotti-Jametti

**Affiliations:** ^1^Department of Neurology, University Hospital Essen, University of Duisburg-Essen, Essen, Germany; ^2^Department of Neurology and Neuroimmunology Unit, San Raffaele Hospital, Milan, Italy; ^3^Department of Clinical Neurosciences, University of Cambridge, Cambridge, United Kingdom; ^4^Department of Metabolism, Digestion and Reproduction, Imperial College London, London, United Kingdom

**Keywords:** neuroplasticity, axonal plasticity, dendritic plasticity, maladaptive plasticity, neuronal regeneration, synaptic plasticity, functional neurological recovery

Clinical neurosciences were heavily influenced by the Spanish neuropathologist Santiago Ramon y Cajal (1852–1934) for ~80 years. Cajal, credited with discovering the axonal growth cone, conducted meticulous silver nitrate stainings, which revealed the ability of adult central nervous system (CNS) neurons to sprout and form new axonal connections during learning and memory processes (Sherrington, 1935). However, Cajal also observed limited axonal growth of neurons from patients who suffered from brain or spinal cord trauma, leading him to conclude that adult human CNS axons have limited regeneration potential (Ramon y Cajal, 1928). This limitation was considered for years as insurmountable and to be responsible for the deficient neurological recovery seen in many CNS-injured patients.

Indeed for a significant period of time following Cajal's work, the concept of neuroregeneration in the adult CNS was largely abandoned. It was in particular in the 1980's that experimental studies reignited interest in the topic. These studies demonstrated that the restricted plasticity of adult mammalian CNS neurons is linked to axonal growth inhibitors released by glial cells, which actively prevent neuronal sprouting (Schwab, 1990). These growth inhibitors are typically proteins or proteoglycans, which in response to CNS injury are temporarily downregulated, allowing for a time-limited axonal outgrowth, which is associated with spontaneous neurological improvements that might be even boosted therapeutically (Schwab, 1990). Indeed, experimental studies have shown that antibody-mediated deactivation of these growth inhibitors or delivery of growth factors can significantly increase axonal sprouting both *in vitro* and *in vivo*. This, in turn, enhances functional neurological recovery in models of spinal cord injury (Schnell and Schwab, 1990; GrandPré et al., 2002) and ischemic stroke (Martino et al., 2011; Reitmeir et al., 2011; Wahl et al., 2014). Due to the complexity of plasticity processes these promising data have not yet translated into clinically applicable treatments for human patients despite considerable efforts made, and several randomized clinical trials have failed to show efficacy in the past (FOCUS Trial Collaboration, 2019; Chabriat et al., 2020). The road to effective neuroregenerative therapies remains challenging, but the understanding of the role of growth inhibitors and enhancers and their therapeutic modification have opened up promising research avenues in the neuroplasticity field.

In an endeavor to identify particularly promising breakthroughs in translational neurosciences, the Cellular Neuropathology section of Frontiers in Cellular Neurosciences has recently introduced the Hot Topics Hub (Hermann, 2020). This initiative aims to discover impactful papers that have landscape-changing potential, broaden imagination horizons, and expand current diagnostic or therapeutic possibilities. Within this hub, the most recent initiative was a Research Topic on treatments that promote neuronal plasticity in the injured CNS (Hermann). Similar to a previous Research Topic that explored the significance of persistent neuroinflammation in chronic neurodegeneration (Hermann et al., 2022), this new topic sought manuscripts that advance our understanding of neurological diseases, surpass existing limitations, and pave the way for therapeutic advancements. The Research Topic invited papers on axonal, dendritic, or synaptic plasticity, utilizing *in vitro* or *in vivo* models of CNS trauma, ischemia, slowly evolving neurodegeneration, infection, cancer, and other relevant contexts. Among the papers submitted, four papers were judged to fulfill these criteria. We shortly outline these papers in the following text.

Synaptic plasticity is promoted by neurotrophic factors, among which brain-derived neurotrophic factor (BDNF), which signals through high affinity tropomyosin receptor kinase B (TrkB), has a particularly long history of studies in models of brain injury in view of its ability to promote functional neurological recovery (Weishaupt et al., 2012). Notably, earlier studies found that BDNF is upregulated in small sensory neurons after peripheral inflammation (Cho et al., 1997). BDNF can function as a neuromodulator that has been shown to modify synaptic transmission from C fibers to dorsal horn neurons, thereby contributing to central sensitization under conditions of inflammatory pain (Kerr et al., 1999). Using transgenic TrkBF616 mice, Martin et al. examined the effect of pharmacogenetic inhibition of TrkB signaling, induced by treatment with 1NM-PP1 (1NMP) in drinking water for 5 days, on formalin-induced inflammatory pain, pain hypersensitivity, and locomotor dysfunction after thoracic spinal contusion. The authors also studied TrkB, ERK1/2, and phosphorylated ERK1/2 expression in the lumbar spinal cord and trunk skin. Their study showed that formalin-induced pain responses were attenuated in 1NMP-treated mice. Weekly assessment of tactile sensitivity with the von Frey test showed that treatment with 1NMP immediately after spinal cord injury (SCI) blocked the development of mechanical hypersensitivity up to 4 weeks post-SCI. Contrastingly, when treatment started 2 weeks after SCI, 1NMP reversibly and partially attenuated hind-paw hypersensitivity. Locomotor scores were improved in the early-treated 1NMP mice compared to late-treated or vehicle-treated SCI mice. 1NMP treatment attenuated SCI-induced increases in TrkB and phosphorylated ERK1/2 levels in the lumbar cord but failed to exert similar effects in the trunk skin. These results highlight the significance of appropriate timing of plasticity-promoting therapies. They also suggest that plasticity promotion in some cases may also have detrimental maladaptive effects, as shown for TrkB activation which leads to spinal pain hypersensitivity and impaired locomotor function when initiated early after SCI.

Besides neurotrophic factors, brain plasticity is regulated by a large variety of additional extracellular signals, some of which got into the focus of research only recently. Among these, the β-galactoside binding protein galectin-3 belonging to the S-type lectin family plays a multifaceted role accelerating cell proliferation and differentiation, regulating cell-cell interactions within the extracellular matrix, and modulating postnatal subventricular gliogenesis (Chip et al., 2016; Al-Dalahmah et al., 2020). Galectin-3 deletion downregulated BDNF and nerve growth factor (NGF), suppressed microglial proliferation and activation, and induced anxiogenic effects in adult healthy wildtype animals (Lalancette-Hébert et al., 2012; Stajic et al., 2019). Galectin-3 deletion also decreased ischemia-induced angiogenesis, inhibited neural progenitor cell proliferation, and increased neuronal cell injury (Lalancette-Hébert et al., 2012; Chip et al., 2016). Additional studies have also unveiled the association between galectin-3 and myelin formation (Pasquini et al., 2011), yet the significance of this finding in clinically relevant disease models was still unclear. In their study, Wang et al. found that galectin-3 knockdown impeded myelin formation, indicated by Olig2/CC1^+^ mature oligodendrocyte numbers, expression of maturation-associated oligodendroglial markers, and myelin thickness and integrity. Recombinant galectin-3 administration by intracerebroventricular injection notably did not have an effect on oligodendrogenesis and myelin formation under physiological conditions, but attenuated cognitive deficits and induced remyelination after perinatal hypoxic-ischemic white matter injury. As a consequence of this remodeling, microglia polarization shifted toward a restorative M2-like phenotype. Altogether, these data confirm that galectin-3 promotes axonal remyelination and contributes to functional recovery in a clinically relevant perinatal hypoxia-ischemia model.

Following seminal studies demonstrating that electrical stimulation delivered through the epidural space could alleviate diffuse chest and abdominal pain via stimulation of the dorsal columns (Shealy et al., 1967), major efforts have been made to promote functional recovery following SCI by electrical stimulation strategies. Functional improvements in select human patients ranged from weight-bearing locomotion to regaining sexual function and neuropathic pain relief (Harkema et al., 2011). While broader applications in SCI patients remain limited, research efforts in the fields of epidural electrical stimulation (EES), peripheral nerve stimulation (PNS) and functional electrical stimulation (FES) have made promising advancements in the recent past, as the review by Dorrian et al. in this Research Topic outlined. Yet, there is still a paucity of mechanistic understanding, limiting our ability to optimize stimulation devices and parameters, or utilize combinatorial treatments to maximize efficacy, as the authors emphasized. This review provides valuable insights into electrical stimulation methods in the context of SCI, and critically discusses cellular and molecular mechanisms suggested in the literature. Key mechanisms that likely contribute to functional improvements induced by electrical stimulation have been highlighted, and gaps in current knowledge have been identified. Thereby, potential research avenues for future studies have been proposed. Without more profound mechanistic studies, clinical progress in the field of electrical stimulation post-SCI will likely not be achieved.

Another example highlighting the complexity of plasticity processes is the development of epilepsy. Epilepsy is a chronic CNS disease associated with high morbidity, for which to date no disease-modifying treatments have been identified. In their review, Jean et al. examined mechanisms of aberrant neuronal networks and plasticity contributing to epileptogenesis. Although the underlying biological mechanism is not clear, scientific evidence indicates that epilepsy is associated with a hyperexcitable synchronous neuronal network and active dendritic spine plasticity. The changes in dendritic spine morphology are related to altered expression of synaptic cytoskeletal proteins, inflammatory molecules, neurotrophic factors, and extracellular matrix signaling. However, it remains to be determined if these aberrant dendritic spine formations lead to neuronal hyperexcitability and abnormal synaptic connections or whether they constitute an underlying mechanism of seizure susceptibility. Focusing on dendritic spine machinery, as a potential target for medications, could limit or reverse the development of epilepsy in a causative way, as the authors emphasized in their review.

Take home messages of this Research Topic are (i) the need of thorough mechanistic studies, which besides offering the mode-of-action framework for plasticity-promoting treatments may disclose surrogate markers for subsequent proof-of-concept trials in human brain-injured patients, and (ii) the importance of the appropriate timing of treatments, which circumvents the risk of maladaptive plasticity resulting in unfavorable outcomes or treatment complications. There are still challenges ahead in the clinical implementation of plasticity-promoting treatments. This Research Topic helped define valuable research avenues to follow.

## Author contributions

DH: Writing—original draft, Writing—review and editing. MB: Writing—review and editing. LP-J: Writing—review and editing.
